# Distinct characteristics of multisystem inflammatory syndrome in children in Poland

**DOI:** 10.1038/s41598-021-02669-2

**Published:** 2021-12-07

**Authors:** Kamila Maria Ludwikowska, Magdalena Okarska-Napierała, Natalia Dudek, Paweł Tracewski, Jacek Kusa, Krzysztof Piotr Piwoński, Aneta Afelt, Dominik Cysewski, Mateusz Biela, Bożena Werner, Teresa Jackowska, Catherine Suski-Grabowski, Miron Bartosz Kursa, Ernest Kuchar, Leszek Szenborn, MOIS CoR Study Group, Marta Barszcz, Elżbieta Berdej-Szczot, Sebastian Brzuszkiewicz, Piotr Buda, Alicja Czajka, Agnieszka Czech, Ewa Czerwińska, Magdalena Figlerowicz, Małgorzata Firek-Pędras, Aneta Gawlik, Ewelina Gowin, Olga Izdebska, Danuta Januszkiewicz-Lewandowska, Justyna Kiepuszka, Agnieszka Koczwara, Danuta Koszałko, Magdalena Kośmider-Żurawska, Janusz Książyk, Beata Kucińska, Martyna Kukawska, Anita Lackowska, Katarzyna Łapacz, Agnieszka Maliszak, Anna Mania, Joanna Mańdziuk, Artur Mazur, Katarzyna Mazur-Melewska, Cezary Niszczota, Paulina Opalińska-Zielonka, Ilona Pałyga-Bysiecka, Katarzyna Rojewska, Anna Rożnowska-Wójtowicz, Bartosz Siewert, Paulina Sobiczewska, Lidia Stopyra, Agnieszka Stroba-Żelek, Joanna Stryczyńska-Kazubska, Tomasz Szatkowski, Barbara Szczepańska, Maciej Szczukocki, Robert Szylo, Filip Tyc, Katarzyna Wielgos, Ewa Wołowska, Jacek Wysocki, Anna Zacharzewska, Marcin Zaniew, Marzena Zielińska, Katarzyna Zięba-Glonek

**Affiliations:** 1 Department of Pediatric Infectious Diseases, Wroclaw Medical University, Chałubińskiego 2-2a, 50-368 Wrocław, Poland; 2 Department of Pediatrics with Clinical Assessment Unit, Medical University of Warsaw, Żwirki i Wigury 61, 02-091 Warsaw, Poland; 3 Department of Pediatric Cardiology, Research and Development Center0, Regional Specialist Hospital in Wroclaw, Kamieńskiego 73a, 51-124 Wrocław, Poland; 4 Interdisciplinary Centre for Mathematical and Computational Modelling, University of Warsaw, Pawinskiego 5A, 02-106 Warsaw, Poland; 5 Espace-DEV, IRD - Institut de Recherche pour le Développement, 500 rue Jean-François Breton, 34393 Montpellier Cedex 05, France; 6 Institute of Biochemistry and Biophysics, Polish Academy of Sciences, Pawinskiego 5A, 02-106 Warsaw, Poland; 7 Department of Paediatrics and Rare Disorders, Wroclaw Medical University, Wrocław, Poland; 8 Department of Pediatric Cardiology and General Pediatrics, Medical University of Warsaw, Żwirki i Wigury 61, 02-091 Warsaw, Poland; 9 Department of Pediatrics, The Medical Centre of Postgraduate Education, Cegłowska 80, 01-809 Warsaw, Poland; 10 Department of Pediatric Infectious Diseases, Medical University of Białystok, Waszyngtona 17, 15-274 Białystok, Poland; 11 Department of Paediatrics and Paediatric Endocrinology, Upper-Silesian Paediatric Health Center School of Medicine in Katowice, Medical University of Silesia, Katowice, Poland; 12 Provincial Specialist Children’s Hospital Prof. S. Popowski in Olsztyn, ul. Żołnierska, 18a, 10-651 Olsztyn, Poland; 13 Department of Pediatrics, Nutrition and Metabolic Diseases, The Children’s Memorial Health Institute, Al. Dzieci Polskich 20, 04-730 Warsaw, Poland; 14 Department of Pediatrics, Children’s Hospital, ul. Niekłańska 4/24, 03-924 Warsaw, Poland; 15 Department of Infectious Diseases and Child Neurology, Poznań University of Medical Science, Szpitalna 27/33, 60-572 Poznan, Poland; 16 Poznań University of Medical Science, Smoluchowskiego 11, 60-179 Poznan, Poland; 17 Clinical Department of Paediatrics and Nephrology, Voivodeship Complex Hospital L. Rydygiera in Toruń, ul. św. Józefa 53-59, 87-100 Toruń, Poland; 18 Department of Pediatric Oncology, Hematology and Transplantation, Poznań University of Medical Sciences, Szpitalna 27/33, 60-572 Poznan, Poland; 19 Specialist Hospital F. Ceynowy, ul. dr. A. Jagalskiego 10, 84-200 Wejherowo, Poland; 20 Department of Pediatrics and Rheumatology, Specialist Hospital Antoniego Falkiewicza, Warszawska 2, 52-114 Wrocław, Poland; 21 Independent Public Health Care, ul. Sukiennicza 13, 64-500 Szamotuły, Poland; 22 Department of Anaesthesiology and Intensive Therapy, Wrocław Medical University, ul. Borowska 213, 50-556 Wrocław, Poland; 23 Clinical Department of Pediatrics, Mazowiecki Specialist Hospital, ul. Aleksandrowicza 5, Radom, Poland; 24 Children’s Hospital “Polanki”, Polanki 119, 80-308 Gdańsk, Poland; 25 Pediatric Ward, Regional Specialist Hospital, ul. Iwaszkiewicza 5, 59-220 Legnica, Poland; 26 Collegium Medicum University of Jan Kochanowski, ul. Grunwaldzka 45, 25-736 Kielce, Poland; 27 Department of Pediatrics, Pediatric Endocrinology and Diabetes, University of Rzeszów, Lwowska 60, 35-301 Rzeszow, Poland; 28 Department of Preventive Health, Poznań University of Medical Science, Smoluchowskiego 11, 60-179 Poznan, Poland; 29 Multidiscyplinary Hospital in Nowa Sól, ul. Chałubińskiego 1, 67-100 Nowa Sól, Poland; 30 Department of Infectious Diseases and Paediatrics, S. Zeromski Hospital in Krakow, Osiedle Na Skarpie 66, 31-913 Kraków, Poland; 31 Chair and Department of Environmental Medicine and Epidemiology, Pediatric Ward, City Hospital in Ruda Śla̧ska, Medical University of Silesia, ul. Wincentego Lipa 2, 41-703 Ruda Slaska, Poland; 32 Congenital Heart Disease and Pediatric Cardiology Department, Silesian Center for Heart Diseases in Zabrze, ul. Marii Curie Skłodowskiej 9, 41-800 Zabrze, Poland; 33 Department of Paediatrics, J. Gromkowski Regional Specialist Hospital in Wrocław, Koszarowa 5, Wrocław, Poland; 34 2nd Infectious Diseases Ward, Children’s Hospital in Poznań, Nowowiejskiego 56/58, 61-734 Poznan, Poland; 35 University Clinical Center: Central Teaching Clinical Hospital, ul. Banacha 1A, 02-097 Warsaw, Poland; 36 Department of Pediatrics, University of Zielona Góra, Zielona Gora, Poland; 37 District Hospital of the name of Doctor T. Chalubinski in Zakopane, ul. Kamieniec 10, Zakopane, Poland

**Keywords:** Infectious diseases, Inflammation, Epidemiology, Immunological disorders, Infectious diseases

## Abstract

During the winter months of 2020/2021 a wave of multisystem inflammatory syndrome in children (MIS-C) emerged in Poland. We present the results of a nationwide register aiming to capture and characterise MIS-C with a focus on severity determinants. The first MIS-C wave in Poland was notably high, hence our analysis involved 274 children. The group was 62.8% boys, with a median age of 8.8 years. Besides one Asian, all were White. Overall, the disease course was not as severe as in previous reports, however. Pediatric intensive care treatment was required for merely 23 (8.4%) of children, who were older and exhibited a distinguished clinical picture at hospital admission. We have also identified sex-dependent differences; teenage boys more often had cardiac involvement (decreased ejection fraction in 25.9% vs. 14.7%) and fulfilled macrophage activation syndrome definition (31.0% vs. 15.2%). Among all boys, those hospitalized in pediatric intensive care unit were significantly older (median 11.2 vs. 9.1 years). Henceforth, while ethnicity and sex may affect MIS-C phenotype, management protocols might be not universally applicable, and should rather be adjusted to the specific population.

## Introduction

Pediatric inflammatory multisystem syndrome temporally associated with severe acute respiratory syndrome coronavirus 2 (SARS-CoV-2) or multisystem inflammatory syndrome in children (herein referred to as MIS-C) is a new entity which has emerged in countries particularly hit by coronavirus disease 2019 (COVID-19) pandemic^1–9^. Despite similarities to other inflammatory conditions, e.g. Kawasaki disease (KD), macrophage activation syndrome (MAS) or toxic shock syndrome, MIS-C has its distinct features^8,9^. MIS-C is characterized by a sudden onset of rapidly progressing multisystem inflammation which particularly affects the cardiovascular system, resulting in cardiac dysfunction and shock. Milder forms of fever and inflammation were also described, however in the largest published cohorts majority of patients necessitated treatment in a pediatric intensive care unit (PICU)^4–11^.

Following COVID-19 second wave in Autumn 2020 (first significant wave in Poland), a rise in MIS-C prevalence has emerged. Here we report the results of a national register of inflammatory disorders in children (MultiOrgan Inflammatory Syndromes COVID-19 Related Study, MOIS-CoR) revealing a consistent picture of MIS-C in our population, yet with some unique local characteristics.

## Results

### Study group

**Figure 1:**
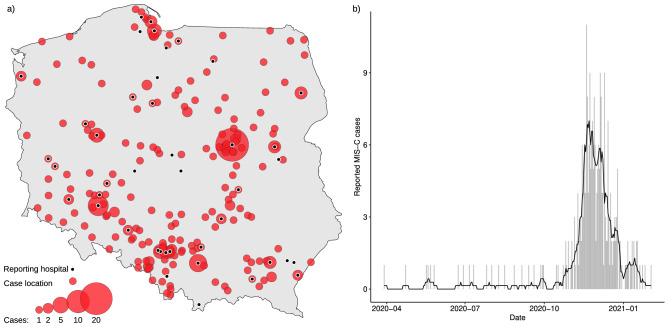
Geospatial and temporal distribution of analyzed cases. (**a**) Shows locations of analyzed cases and reporting sentinel sites. (**b**) Shows the counts MIS-C hospitalisations in respective days in the analyzed period, between 4th March 2020 and 20th February 2021. Bars indicate daily counts, while the black line presents the 7-day moving average.

Children aged 0–18 years old with inflammatory conditions were voluntarily reported by treating clinicians. The surveillance was launched on 25th May 2020, and, as of 20th February 2021, 399 children have been reported from 42 cities all over the country (Fig. 1). 342 cases fulfilled the inclusion criteria and 274 fulfilled MIS-C diagnostic criteria (Table 1, Supplementary Table 3). The following results apply only to the cohort of 274 children, aged from 7 months to 17.6 years, with MIS-C. Precise demographic characteristics of the MIS-C cohort are presented in Table 1. All but one Asian child were of White ethnicity, 172 (62.8%) were male. A minority of children had any comorbidities, with obesity (6.7%), asthma (4.1%) and neurological disorders (2.3%) being the most prevalent.

**Table 1:** Demographic characteristic and SARS-CoV-2 status of a studied group.

	All (399)	MIS-C (274)
Male sex	250 (63%)	171 (62%)
Age (years)	7.8 (3.9–11.5)	8.8 (5.2–12.1)
**Ethnicity**		
White	397 (99%)	273 (100%)
Asian	2 (1%)	1 (0%)
**Comorbidity**	58 (19%)	38 (18%)
Asthma	13 (3%)	11 (4%)
Obesity	29 (8%)	16 (7%)
Neurological disorder	7 (2%)	6 (2%)
Immunosuppression	3 (1%)	1 (0%)
BMI Z-score	0.0 (− 0.8 to 0.9)	0.1 (− 0.8 to 0.9)
**SARS-CoV-2 tests and anamnesis**		
Contact with a confirmed COVID-19 case	144 (45%)	120 (52%)
Confirmed preceding SARS-CoV-2 infection	30 (9%)	24 (10%)
Positive SARS-CoV RT-PCR result	41 (12%)	29 (13%)
Positive antibodies against SARS-CoV-2 at admission	262 (80%)	241 (95%)

*Binary data given as count (per-cent), and numerical data as median (interquartile range).*

*BMI﻿* body mass index, *COVID-19* coronavirus disease 2019, *RT-PCR* real-time transcription polymerase chain reaction, *SARS-CoV-2* severe acute respiratory syndrome coronavirus 2.

### Clinical presentation

The median time between first symptoms and hospital admission was 5 days, and the median fever length was 7 days. The vital signs at admission and at their respective peaks are presented in Table 2 and Supplementary Table 5. Complete clinical characteristics of children with MIS-C are presented in Table 3 and Supplemental Table 4.

**Table 2:** Vital signs and laboratory results of MIS-C cohort at admission and at respective peaks.

	All (274)	PICU (23)	Non-PICU (251)	P	P|Age	P|Sex
CRT > 2 s adm	30 (13%)	8 (40%)	22 (11%)	< 0.01	< 0.01	< 0.01
CRT max > 2 s	32 (15%)	10 (56%)	22 (11%)	< 0.01	< 0.01	< 0.01
Saturation adm [%]	98.0 (96.0–99.0)	97.0 (95.0–98.0)	98.0 (96.0–99.0)	0.09	0.08	0.09
Saturation min [%]	96.0 (92.0–98.0)	92.0 (89.8–97.0)	96.0 (93.0–98.0)	0.02	0.04	0.02
Respiratory rate adm [1/min]	20.0 (18.0–25.0)	30.0 (20.0–45.0)	20.0 (18.0–25.0)	0.01	< 0.01	< 0.01
Respiratory rate max [1/min]	25.0 (20.0–30.0)	40.0 (30.0–49.5)	24.0 (20.0–30.0)	< 0.01	< 0.01	< 0.01
Heart rate adm [1/min]	120.0 (100.0–133.0)	127.0 (110.0–140.0)	120.0 (100.0–133.0)	0.3	0.08	0.2
Heart rate max [1/min]	132.5 (118.0–150.0)	135.0 (120.0–160.0)	132.0 (118.0–146.5)	0.2	0.06	0.2
SBP adm [mmHg]	100.0 (90.0–110.0)	89.0 (78.8–99.5)	100.0 (91.0–110.0)	< 0.01	< 0.01	< 0.01
SBP min [mmHg]	88.0 (78.0–96.0)	74.0 (62.5–82.5)	89.0 (80.0–96.8)	< 0.01	< 0.01	< 0.01
Non-alert AVPU adm	17 (6%)	5 (22%)	12 (5%)	< 0.01	< 0.01	< 0.01
Non-alert AVPU min	38 (15%)	8 (44%)	30 (13%)	< 0.01	< 0.01	< 0.01
WBC adm [$10^9$/l]	9.6 (6.6–13.3)	11.6 (7.1-19.1)	9.6 (6.6-12.8)	0.1	0.1	0.1
WBC min [$10^9$/l]	6.7 (5.0–9.0)	6.0 (5.3–9.2)	6.7 (5.0–9.0)	0.9	0.8	0.9
WBC max [$10^9$/l]	14.5 (10.9–19.7)	19.8 (13.1–28.4)	14.3 (10.5–19.1)	< 0.01	< 0.01	< 0.01
Lymphocytes adm [$10^9$/l]	1.0 (0.7–1.8)	0.8 (0.6–1.0)	1.1 (0.7–1.8)	0.05	0.3	0.05
Lymphocytes min [$10^9$/l]	1.0 (0.6–1.8)	0.6 (0.5–0.9)	1.0 (0.6–1.8)	< 0.01	0.07	< 0.01
Hemoglobin adm [g/dl]	11.7 (10.7–12.7)	11.1 (10.3–12.1)	11.8 (10.7–12.7)	0.1	0.02	0.1
Hemoglobin min [g/dl]	10.3 (9.4–11.2)	9.7 (8.8–10.7)	10.3 (9.4–11.2)	0.06	< 0.01	0.05
Platelets adm [$10^9$/l]	176.0 (127.0–248.0)	153.0 (121.0–187.5)	178.0 (128.5–255.8)	0.08	0.1	0.07
Platelets min [$10^9$/l]	160.0 (109.8–230.8)	135.5 (93.8–182.5)	163.5 (111.0–243.2)	0.08	0.09	0.06
CRP adm [mg/l]	140.0 (83.7–194.9)	242.0 (123.3–289.0)	133.4 (78.8–187.2)	< 0.01	< 0.01	< 0.01
CRP max [mg/l]	166.3 (94.4—242.1)	264.6 (206.7–309.4)	161.4 (93.3–226.2)	< 0.01	< 0.01	< 0.01
Procalcitonin adm [ng/ml]	2.5 (1.0–6.9)	13.2 (2.1–51.2)	2.3 (0.9–6.2)	< 0.01	< 0.01	< 0.01
Procalcitonin max [ng/ml]	4.3 (1.3–12.9)	17.0 (10.2–30.7)	3.5 (1.2–10.0)	< 0.01	< 0.01	< 0.01
ESR adm [mm]	44.0 (31.0–66.0)	78.5 (73.0–81.2)	44.0 (30.0–65.0)	0.03	0.02	0.02
ESR max [mm]	57.0 (37.8–80.0)	77.0 (52.5–82.5)	55.0 (37.0–78.0)	0.4	0.4	0.4
Ferritin adm [ng/ml]	331.0 (197.9–622.4)	671.0 (475.9–1052.8)	317.1 (186.2–533.8)	< 0.01	< 0.01	< 0.01
Ferritin max [ng/ml]	402.2 (217.9–672.2)	671.0 (559.2–1113.4)	367.9 (207.3–616.5)	< 0.01	< 0.01	< 0.01
Triglycerides adm [mg/dl]	147.0 (123.0–218.9)	181.0 (148.5–266.5)	145.0 (119.0–213.0)	0.05	0.04	0.05
Triglycerides max [mg/dl]	172.5 (129.0–256.2)	194.0 (169.0–359.0)	167.0 (125.0–246.0)	0.07	0.05	0.07
D-dimers adm [mg/l]	2.6 (1.5–4.6)	3.9 (2.5–5.7)	2.5 (1.4–4.4)	0.01	< 0.01	< 0.01
D-dimers max [mg/l]	3.8 (2.0–6.3)	5.7 (3.6–8.2)	3.7 (2.0–6.2)	0.03	0.05	0.02
AlAT adm [U/l]	24.0 (16.0–40.1)	35.0 (16.0–92.0)	23.0 (15.5–40.0)	0.05	0.06	0.05
AlAT max [U/l]	33.3 (21.0–65.8)	55.0 (32.0–122.0)	33.0 (20.0–59.2)	< 0.01	0.03	< 0.01
AST adm [U/l]	32.1 (25.0–51.5)	37.0 (25.0–59.0)	32.0 (24.5–51.0)	0.3	0.3	0.3
AST max [U/l]	43.0 (30.0–65.0)	57.0 (44.0–108.0)	42.0 (30.0–63.5)	< 0.01	0.01	< 0.01
CK adm [U/l]	51.0 (37.0–92.0)	22.4 (13.2–30.9)	52.0 (38.0–93.0)	0.01	< 0.01	0.02
CK max [U/l]	51.0 (37.0–85.0)	27.9 (24.0–38.0)	56.5 (37.2–92.8)	0.01	< 0.01	0.01
Sodium adm [mmol/l]	135.0 (132.0–137.0)	134.0 (132.0–137.8)	135.0 (132.0–137.0)	1	0.9	0.8
Sodium min [mmol/l]	133.6 (131.0–135.9)	133.0 (130.0–135.0)	133.8 (131.0–135.9)	0.5	0.6	0.6
Albumins adm [g/dl]	3.3 (2.8–3.7)	2.8 (2.4–3.4)	3.3 (2.8–3.7)	0.02	< 0.01	0.01
Albumins min [g/dl]	2.8 (2.5–3.3)	2.5 (2.3–2.7)	2.8 (2.5–3.3)	< 0.01	< 0.01	< 0.01
Troponin elevated adm	62 (28%)	17 (77%)	45 (22%)	< 0.01	< 0.01	< 0.01
Troponin elevated max	92 (51%)	18 (86%)	74 (47%)	< 0.01	< 0.01	< 0.01
BNP/NT-proBNP elevated adm	171 (86%)	16 (89%)	155 (85%)	0.7	0.6	0.7
BNP/NT-proBNP elevated max	204 (91%)	20 (100%)	184 (90%)	0.1	0.2	0.1
eGFR adm [ml/min/1.73 m$^2$]	110.1 (86.2–134.3)	77.1 (50.8–89.5)	113.1 (90.5–134.8)	< 0.01	< 0.01	< 0.01
eGFR min [ml/min/1.73 m$^2$]	104.9 (82.0–126.6)	73.0 (45.4–88.0)	107.4 (85.4–128.8)	< 0.01	< 0.01	< 0.01

Binary data given as count (per-cent), and numerical data as median (interquartile range). Values at admission are marked with adm, lowest obtained with min, while highest with max. Troponin is considered elevated at > 50 ng/l, while BNP/NT-proBNP at > 150 ng/ml.

*AlAT*, alanine transaminase, *AST*, aspartate transaminase, *AVPU* AVPU scale, *BNP/NT-proBNP* brain natriuretic peptide or N-terminal-pro-BNP, *CK* creatinine kinase, *CRT* capillary refill time, *CRP*, C-reactive protein, *eGFR* estimated glomerular filtration rate, *ESR*, erythrocyte sedimentation rate, *MIS-C* multisystem inflammatory syndrome in children, *NT-proBNP* N-terminal prohormone of brain natriuretic peptide, *PICU*, pediatric intensive care unit, *SBP* systolic blood pressure, *WBC* white blood cell count, *P|Age* age-adjusted p-value, *P|Sex* sex-adjusted p-value.

**Table 3:** Demographic and clinical characteristics of MIS-C cohort respective of age.

	All (274)	0–5 y.o. (64)	5–12 y.o. (140)	12–18 y.o. (70)	P	P|Sex
Male sex	171 (62%)	30 (47%)	91 (65%)	50 (71%)	< 0.01	–
**Symptoms and signs**						
Fever length	7.0 (6.0–9.0)	7.0 (6.0–8.0)	7.5 (6.0–9.0)	7.0 (6.0–9.0)	0.3	0.3
Mucocutaneous and lymph nodes	262 (97%)	59 (95%)	138 (99%)	65 (94%)	0.2	0.2
Rash	218 (83%)	53 (84%)	118 (87%)	47 (71%)	0.02	0.03
Conjunctivitis	207 (78%)	47 (76%)	109 (81%)	51 (75%)	0.6	0.5
Hands/feet swelling or erythema	142 (55%)	39 (64%)	75 (56%)	28 (45%)	0.1	0.2
Oral inflammation	173 (66%)	43 (68%)	96 (72%)	34 (52%)	0.02	0.02
Cervical lymphadenopathy	98 (38%)	30 (48%)	46 (36%)	22 (32%)	0.1	0.06
Gastrointestinal	250 (93%)	56 (90%)	132 (95%)	62 (90%)	0.3	0.3
Abdominal pain	222 (85%)	42 (76%)	120 (88%)	60 (87%)	0.1	0.2
Nausea	162 (62%)	28 (47%)	100 (75%)	34 (51%)	< 0.01	< 0.01
Diarrhea	164 (62%)	36 (59%)	84 (61%)	44 (65%)	0.8	1
Lower respiratory	128 (50%)	23 (40%)	64 (48%)	41 (63%)	0.04	0.04
Chest pain	48 (19%)	3 (6%)	19 (14%)	26 (39%)	< 0.01	< 0.01
Cough	74 (28%)	17 (28%)	33 (25%)	24 (35%)	0.3	0.2
Breathing effort	63 (24%)	8 (13%)	36 (27%)	19 (28%)	0.08	0.1
Neurological	212 (82%)	52 (84%)	109 (81%)	51 (80%)	0.8	0.9
Lethargy	151 (59%)	36 (61%)	88 (66%)	27 (44%)	0.02	0.01
Irritability	108 (42%)	40 (65%)	52 (39%)	16 (25%)	< 0.01	< 0.01
Headache	112 (46%)	16 (32%)	67 (51%)	29 (47%)	0.07	0.04
Meningeal signs	27 (10%)	6 (10%)	15 (11%)	6 (9%)	0.9	0.8
Cardiovascular	139 (58%)	16 (33%)	79 (63%)	44 (69%)	< 0.01	< 0.01
Hypotension	99 (41%)	7 (14%)	57 (46%)	35 (51%)	< 0.01	< 0.01
Coronary dilation or aneurysm	21 (8%)	6 (11%)	10 (7%)	5 (8%)	0.8	0.5
EF < 55%	58 (23%)	3 (5%)	31 (23%)	24 (39%)	< 0.01	< 0.01
Musculo-osteoarticular	111 (44%)	17 (32%)	58 (44%)	36 (55%)	0.04	0.03
Muscle pain	103 (41%)	15 (29%)	53 (40%)	35 (54%)	0.02	0.01
Arthralgia	50 (19%)	6 (10%)	29 (21%)	15 (24%)	0.1	0.08
Arthritis	12 (5%)	1 (2%)	6 (4%)	5 (8%)	0.2	0.2
**Clinical presentation**						
KD or aKD	170 (67%)	43 (70%)	94 (71%)	33 (54%)	0.06	0.08
KD	105 (42%)	29 (48%)	57 (44%)	19 (31%)	0.1	0.2
MAS	59 (22%)	4 (6%)	30 (22%)	25 (36%)	< 0.01	< 0.01
DIC	42 (17%)	5 (9%)	22 (17%)	15 (22%)	0.1	0.06

Binary data given as count (per-cent), and numerical data as median (interquartile range).

*BMI* body mass index, *DIC* disseminated intravascular coagulation, *EF* ejection fraction, *KD* Kawasaki disease, *MAS* macrophage activation syndrome, *MIS-C* multisystem inflammatory syndrome in children, *y.o.* years old, *P|Sex* sex-adjusted p-value. Hypotension defined as minimal SBP in mmHg less than $70 + 2 \times$ age in years or 90 for children over 10 years old.

Mucocutaneous and lymph node involvement was observed in 95.6% of children, and 66.7% fulfilled American Heart Association (AHA) KD or atypical KD (aKD) diagnostic criteria, irrespective of age. Rash was less common in children >12 years of age (71.2% vs. 86.4%, p < 0.01), whereas conjunctival injection, distal extremity changes and cervical lymphadenopathy were equally prevalent across age groups. The second most common group of associating symptoms was gastrointestinal (92.6%), with no significant differences across age groups, except for nausea and vomiting, which were more prevalent in children 5–12 years of age (74.6% vs. 48.8%, p < 0.01). Ten children underwent abdominal surgery due to acute abdominal symptoms.

Hypotension was present in 30/213 (14.1%) of patients at admission, while 99/243 (40.7%) developed it at some point during hospitalization. At least one echocardiogram was reported for 255 children, of whom 85 (33.3%) had any of the following abnormalities: decreased ejection fraction (EF), coronary artery abnormality (CAA) or pericardial effusion. EF < 55% was reported in 58/255 (22.7%) of children (four had EF < 35%), and the age of children with heart dysfunction overall was significantly higher (median [IQR] from 7.9 [4.9–11.3] to 11.0 years [8.8–13.6], p < 0.01). CAA developed in 21/255 (8.2%) of patients irrespective of age or KD or aKD phenotype. In particular, eight children developed coronary artery aneurysms; three of them resolved in the follow up before discharge. Pericardial effusion was present in 24/255 (9.4%) of reports.

Neurological symptoms included lethargy (59.4%), irritability (41.7%), headache (46.1%) and photophobia (11.0%). Children >12 years old less frequently presented lethargy (44.3% vs. 64.2%, p < 0.01) and irritability (25.0% vs. 47.2%, p < 0.01). Meningeal signs were observed in 27/260 (10.4%) of cases, irrespective of age, and six patients had aseptic meningitis.

Respiratory symptoms included sore throat (34.0%), cough (28.1%) and dyspnoea (24.1%), and they were uniformly prevalent across age groups. Patients >12 years old more often complained of chest pain (39.4% vs. 11.8%, p < 0.01) and muscle pain (53.8% vs. 36.6%, p = 0.01).

Dysuria was reported in 40/257 (15.6%) of children, while sterile leukocyturia in 48/247 (19.4%).

### Laboratory results

Laboratory parameters at admission and at respective peaks are summarized in Table 2. Lymphopenia was present in 195/269 (72.5%) of children, and 148/269 (55.0%) developed lymphopenia of below $1.0\times 10^9$/l. Anemia was present in 254/273 (93.0%) of patients. Thrombocytopenia occurred in 131/273 (48.0%) of children, and 31/273 (11.4%) had a platelet count below $80\times 10^9/$l.

Following MIS-C case definition, all children had increased inflammatory markers: 158/266 (59.4%) had procalcitonin >2.5 ng/ml and 157/273 (57.5%) had C-reactive protein (CRP) >150 mg/l. Hypoalbuminemia was present in 219/260 (84.2%) of patients, and in 71/260 (27.3%) albumin level fell under 2.5 g/dl. Hyponatremia was found in 203/272 (74.6%) of patients, and in 9/272 (3.3%) it was < 125 mmol/l.

Finally, 62/222 (27.9%) of patients had elevated troponin at admission, and this ratio increased to 51.4% when assessed later in course of the disease. Children with elevated troponin levels at admission were older (median [IQR] from 7.9 [4.9–10.7] to 11.4 years [9.0–13.5], p < 0.01), and the same observation held for troponin level at its peak (median [IQR] from 8.5 [5.0–11.9] to 11.2 years [8.7–13.4], p < 0.01).

### Sex-dependent clinical and laboratory characteristics

**Figure 2:**
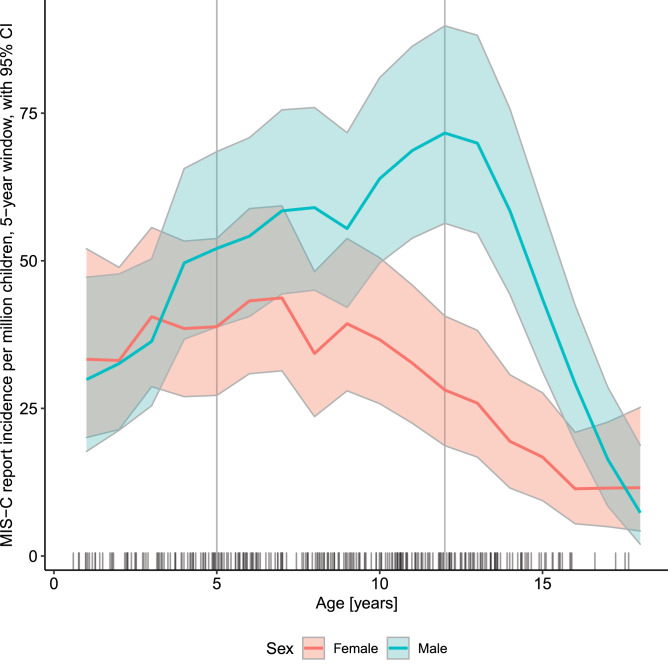
Incidence of reported MIS-C cases within the Polish population of children aged 0–18 years, according to age and sex.

**Figure 3:**
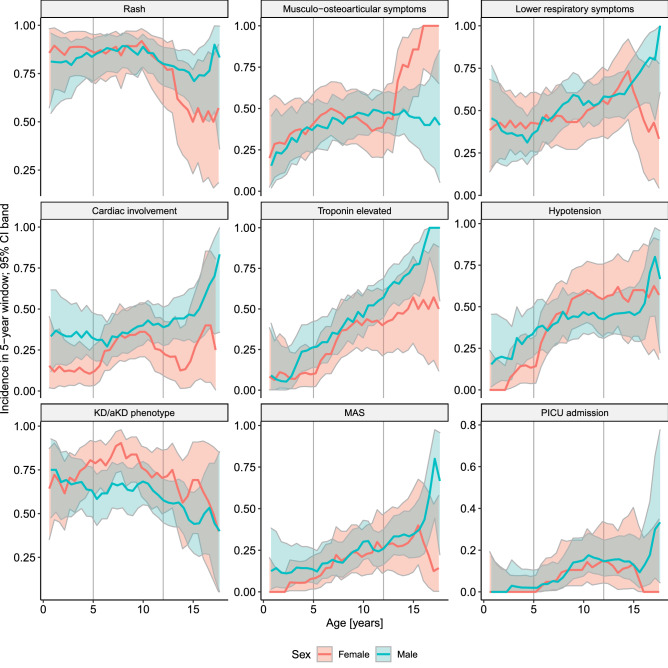
Incidence of the selected events within the study group, according to age and sex. KD/aKD, Kawasaki disease/atypical Kawasaki disease; MAS, macrophage activation syndrome. Musculo-osteoarticular symptoms encompassed: muscle pain, arthralgia, or arthritis. Lower respiratory symptoms encompassed: chest pain, cough, or dyspnea. Cardiac involvement encompassed left ventricular ejection fraction < 55%, or coronary artery abnormalities (dilation or aneurysm), or pericardial effusion.

Male patients were diagnozed with MIS-C more often than expected from demographic structure, but only in the older age bracket (Fig. 2). We have identified some characteristics which corresponded with this discrepancy (Fig. 3, Table 3).

Teenage boys over 12 years old more prevalently had cardiac involvement (25.9% vs. 14.7%, p = 0.03) and fulfilled MAS diagnostic criteria more often (31.0% vs. 15.2%, p < 0.01). Among all boys, those hospitalised in PICU were significantly older (median [IQR] from 9.1 [5.7–12.3] to 11.2 years [10.3–12.6], p=0.02), while there was no such trend for girls.

On the other hand, teenage girls more frequently presented osteoarticular and muscular symptoms (12.6% vs. 2.9%, p < 0.01), but less frequently rash (5.0% vs. 15.2%, p=0.01). KD/aKD phenotype prevalence did not differ between reported girls and boys of any age.

### Pediatric intensive care treatment

Statistics regarding PICU treatment are presented in Tables 4 and  5. PICU treatment was required in 23/274 (8.4%) of children, ten of whom were mechanically ventilated. There were no children treated with extracorporeal membrane oxygenation (ECMO), neither with renal replacement therapy. Two deaths were reported: one in a severely immunocompromised child, and one in previously healthy teenager with fulminant multiorgan dysfunction, both with positive real-time polymerase chain reaction (RT-PCR) test result for SARS-CoV-2. In either case, it was impossible to determine whether the cause of death was a cytokine storm due to COVID-19 or MIS-C; they fulfilled the MIS-C criteria though, and hence were included in this analysis.

**Table 4:** Demographic and clinical characteristics of MIS-C cohort respective of pediatric intensive care involvement.

	All (274)	PICU (23)	Non-PICU (251)	P	P|Age	P|Sex
Male sex	171 (62%)	17 (74%)	154 (61%)	0.2	0.4	1
Age (years)	8.8 (5.2–12.1)	11.2 (10.1–12.6)	8.4 (5.0–11.9)	< 0.01	0.03	< 0.01
BMI Z-score	0.1 (− 0.8 to 0.9)	0.7 (− 0.3 to 0.9)	0.0 (− 0.8 to 0.9)	0.2	0.3	0.2
Any comorbidity	38 (18%)	4 (21%)	34 (18%)	0.7	0.8	0.7
**Symptoms and signs**						
Fever length	7.0 (6.0–9.0)	8.0 (7.0–10.0)	7.0 (6.0–9.0)	0.03	0.06	0.04
Mucocutaneous and lymph nodes	262 (97%)	22 (96%)	240 (97%)	0.8	0.8	0.7
Rash	218 (83%)	15 (71%)	203 (84%)	0.2	0.2	0.1
Conjunctivitis	207 (78%)	14 (67%)	193 (79%)	0.2	0.2	0.2
Hands/feet swelling or erythema	142 (55%)	15 (68%)	127 (54%)	0.2	0.1	0.1
Oral inflammation	173 (66%)	14 (67%)	159 (66%)	0.9	0.8	0.9
Cervical lymphadenopathy	98 (38%)	7 (33%)	91 (38%)	0.7	0.9	0.6
Gastrointestinal	250 (93%)	21 (91%)	229 (93%)	0.8	0.8	0.6
Abdominal pain	222 (85%)	21 (91%)	201 (85%)	0.4	0.5	0.5
Nausea	162 (62%)	16 (70%)	146 (61%)	0.4	0.5	0.5
Diarrhea	164 (62%)	18 (78%)	146 (60%)	0.09	0.1	0.1
Lower respiratory	128 (50%)	18 (86%)	110 (47%)	< 0.01	< 0.01	< 0.01
Chest pain	48 (19%)	5 (25%)	43 (18%)	0.5	1	0.5
Cough	74 (28%)	7 (32%)	67 (28%)	0.7	0.8	0.7
Breathing effort	63 (24%)	14 (67%)	49 (20%)	< 0.01	< 0.01	< 0.01
Neurological	212 (82%)	20 (95%)	192 (80%)	0.09	0.08	0.08
Lethargy	151 (59%)	19 (90%)	132 (57%)	< 0.01	< 0.01	< 0.01
Irritability	108 (42%)	6 (27%)	102 (43%)	0.2	0.4	0.2
Headache	112 (46%)	8 (38%)	104 (47%)	0.4	0.3	0.5
Meningeal signs	27 (10%)	2 (9%)	25 (11%)	0.8	0.8	0.9
Cardiovascular	139 (58%)	22 (100%)	117 (54%)	< 0.01	< 0.01	< 0.01
Hypotension	99 (41%)	18 (86%)	81 (36%)	< 0.01	< 0.01	< 0.01
Coronary dilation or aneurysm	21 (8%)	3 (14%)	18 (8%)	0.3	0.3	0.4
EF < 55%	58 (23%)	14 (64%)	44 (19%)	< 0.01	< 0.01	< 0.01
Musculo-osteoarticular	111 (44%)	8 (42%)	103 (44%)	0.8	0.6	0.9
Muscle pain	103 (41%)	8 (42%)	95 (41%)	0.9	0.8	0.9
Arthralgia	50 (19%)	4 (20%)	46 (19%)	0.9	0.8	0.9
Arthritis	12 (5%)	1 (5%)	11 (5%)	1	0.8	1
**Clinical presentation**						
KD or aKD	170 (67%)	18 (86%)	152 (65%)	0.05	0.03	0.04
KD	105 (42%)	10 (50%)	95 (41%)	0.4	0.3	0.3
MAS	59 (22%)	9 (43%)	50 (20%)	0.02	0.08	0.02
DIC	42 (17%)	5 (22%)	37 (16%)	0.5	0.7	0.4

Binary data given as count (per-cent), and numerical data as median (interquartile range).

*BMI* body mass index, *DIC* disseminated intravascular coagulation, *EF* ejection fraction, *KD*, Kawasaki disease, *MAS* macrophage activation syndrome, *MIS-C* multisystem inflammatory syndrome in children, *PICU* pediatric intensive care unit, *y.o.* years old; *P|Age* age-adjusted p-value, *P|Sex*, sex-adjusted p-value. Hypotension defined as minimal SBP in mmHg less than $70+2\times$ age in years or 90 for children over 10 years old.

**Table 5:** Therapy and outcome of MIS-C cohort respective of age.

	All (274)	0–5 y.o. (64)	5–12 y.o. (140)	12–18 y.o. (70)	P	P|Sex
Admission since onset (days)	5.0 (4.0–6.0)	5.0 (4.0–5.0)	5.0 (4.0–6.0)	5.0 (4.0–6.5)	0.5	0.6
Intensive care	23 (8%)	1 (2%)	13 (9%)	9 (13%)	0.05	0.07
Mechanical ventilation	10 (4%)	–	7 (5%)	3 (5%)	0.2	0.2
Oxygen supplementation	61 (23%)	5 (8%)	41 (31%)	15 (23%)	< 0.01	< 0.01
No immunomodulatory agent	8 (3%)	3 (5%)	3 (2%)	2 (3%)	0.6	0.4
IVIG	238 (93%)	55 (90%)	122 (95%)	61 (91%)	0.3	0.3
GCS	143 (67%)	27 (56%)	74 (69%)	42 (71%)	0.2	0.2
IVIG and GCS	133 (62%)	24 (50%)	69 (65%)	40 (67%)	0.1	0.2
**Any other immunomodulator**						
Tocilizumab	1 (0%)	1 (2%)	–	–	0.2	0.1
Cyclosporin A	2 (1%)	–	2 (1%)	–	0.4	0.3
ASA	226 (85%)	51 (81%)	119 (88%)	56 (84%)	0.4	0.4
Heparin	91 (38%)	14 (25%)	45 (38%)	32 (49%)	0.02	0.02
Warfarin	1 (0%)	1 (2%)	–	–	0.2	0.1
Complete recovery at discharge$^*$	218 (93%)	55 (95%)	115 (94%)	48 (87%)	0.2	0.2

Binary data given as count (per-cent), and numerical data as median (interquartile range).

*ASA* acetylsalicylic acid, *GCS* glucocorticoids, *IVIG* intravenous immunoglobulin, *y.o.* years old, *P|Sex* sex-adjusted p-value.

*All signs and symptoms resolved at discharge.

Children who were hospitalised in PICU were significantly older (median [IQR] from 8.4 [5.0–11.9] to 11.2 years [10.1–12.6], p < 0.01). The PICU admission rate did not significantly differ for patients with comorbidities, including obesity (Table 4, Supplementary Table 4). The median time of hospital admission since the first symptoms was 5 days and it was not significantly different in children admitted to PICU versus the others. At admission, chil×dren who necessitated intensive care, had higher respiratory rate (from median [IQR] 20.0 [18.0–25.0] to 30.0/min [20.0–45.0], p = 0.01) and lower systolic blood pressure (SBP) (from median [IQR] 89.0 [78.8–99.5] to 100.0 mmHg [91.0–110.0], p < 0.01), despite their higher age. They were also more likely to have prolonged capillary refill time (CRT) (40.0% vs. 10.8%, p < 0.01) and AVPU scale score below A (21.7% vs. 5.0%, p < 0.01). PICU-hospitalized children had also significantly higher CRP (from median [IQR] 133.4 [78.8–187.2] to 242.0 mg/dl [123.3–289.0], p < 0.01), procalcitonin (from median [IQR] 2.3 [0.9–6.2] to 13.2 ng/ml [2.1–51.2], p < 0.01), ferritin (from median [IQR] 317.1 [186.2–533.8] to 671.0 $\upmu$g/l [475.9–1052.8], p < 0.01), D-dimers (from median [IQR] 2.5 [1.4–4.4] to 3.9 $\upmu$g/ml [2.5–5.7], p = 0.01), lower estimated glomerular filtration rate (eGFR) (from median [IQR] 77.1 [50.8–89.5] to 113.1 [90.5-134.8], p < 0.01) and more commonly elevated troponin (77.3% vs. 22.5%, p < 0.01). In the later course, children in PICU more commonly had lymphopenia (81.0% vs. 47.0%, p < 0.01), and hypoalbuminemia (100.0% vs. 81.0%, p=0.04).

## Discussion

### Prevalence of MIS-C in Poland

Our study supports current knowledge about MIS-C. Notably, this single-country observation is also one of the most numerous European MIS-C cohorts. The number of 274 MIS-C cases captured in Poland with a 7.31 million children population and 2060 cases reported at the same time in the United States of America (USA) with 74 million children^1,12^ suggest that MIS-C prevalence could have reached a comparable level in both countries. The number of reported COVID-19 cases per 100,000 inhabitants in the USA was at that time approximately twice as high as in Poland^13^.

Moreover, Payne estimated the incidence of MIS-C as approximately 9-fold higher among Black and Hispanic or Latino Americans than among White Americans in general population and this trend sustained (with slightly lower rates) among SARS-CoV-2 infected children^14^. In this context, the high number of cases captured in Poland is even more surprising, as all but one child were White.

### Age and sex-related differences in MIS-C presentation

The mucocutaneous, gastrointestinal or respiratory manifestations and laboratory picture in our cohort were more uniform across age groups than in others^4–6,8–10,15,16^. Cardiovascular involvement significantly increased with age however, which is in line with Dufort and Belay findings^10,17^. In opposition to Payne^14^ we found sex-related differences in MIS-C prevalence—higher for boys, but only from pubertal age. Teenage boys also more commonly had cardiac involvement, fulfilled MAS definition and required PICU hospitalisation. More severe course of COVID-19 in adult males is well established^12^. While some authors postulate that it is linked to genetic and immunological background^18^, others suggest that sex hormones play a role^19,20^. COVID-19 and MIS-C are separate entities, but share some similarities being hyperinflammatory conditions. In our cohort, the sex-related differences appeared from pubertal age, which might support the hormonal theory.

### Kawasaki-like disease as the most common presentation of MIS-C

The exact pre-pandemic incidence of KD in Poland was not known (there had been no national reporting effort before the pandemic), though it was not likely to substantially differ from other European countries^21^. Similarly as in other countries^7,22,23^, we have observed a sharp increase in KD cases following COVID-19 wave. The data limited to two reporting sites supporting these observations are presented in Supplementary Table 6. Despite being initially reported as Kawasaki-like disease, MIS-C appeared to be a distinct entity soon after^8,9,23–25^. Children in our cohort, more frequently than in others, fulfilled KD/aKD diagnostic criteria but concomitantly presented unique features typical for MIS-C^7,15–17,26,27^.

More prevalent KD/aKD phenotype in our cohort could be explained in several ways. Firstly, we have included both: typical and atypical KD presentations. In previous studies which included both clinical variants, KD prevalence was two times more common than in those in which only typical KD definition was used^15–17,27^. Moreover, the comparison of patients with KD from pre-pandemic and pandemic periods in Spain revealed more frequent atypical presentation (71%) among patients with documented SARS-CoV-2 history^23^. Secondly, KD/aKD phenotype could have been over-represented due to our inclusion criteria and the fact, that the cases were identified and reported by pediatricians, who were more familiar with recognising KD than either TSS, MAS or new inflammatory syndrome. Thirdly, once in the database, the fulfillment of the KD/aKD criteria was verified by the dedicated software, not by clinicians. Thus, we captured cases of atypical presentation that might had been overlooked if identified by clinician’s diagnosis only (as in other reports^10,11,15–17^). Fourthly, some distinct clinical features of our cohort e.g., more prevalent KD phenotype, could be due to specific, homogeneous ethnic background, differing from all other cohorts^10,11,16,26,27^. Clinical phenotype of MIS-C has not been analyzed in relation to ethnicity/race thus far and needs to be further studied.

### Milder course of MIS-C in Poland

In Western Europe and the USA more than half of children with MIS-C required intensive care^10,11,15–17,27^, contributing to a multifold increase in the PICU admissions number^7^. Approximately 1.4–3% of children with MIS-C died^7,10,16,28^. In our study, the course of the disease appeared substantially milder—only 8.4% of patients were hospitalised in PICU and two deaths were reported. Treatment used in our cohort did not differ substantially from other reports—most children received IVIG and a large proportion also got steroids. Fewer children required more than two different immunomodulatory agents^4–9,15–17,27,29^. Moreover, the median day of hospital admission since the first symptoms was similar to other reports^6,17^. Hence, the therapeutic approach is an unlikely factor responsible for more favourable outcome among Polish children with MIS-C.

The data about cardiovascular complications from previous reports are inconsistent. This is partially due to different (sometimes unspecified) definitions used by authors^4,6,17,30^, and various inclusion criteria—either broader than World Health Organization (WHO) MIS-C case definition^30^, or narrowed only to the most severe cases^7^. Decreased SBP, occasionally defined as a shock, was reported in 36–86% of patients with MIS-C, whereas heart failure—in 20–45%^4–8,10,30^. Our findings place Polish children with MIS-C within the “milder end” of the acute cardiovascular complications spectrum described above. Similarly, the prevalence of coronary artery involvement in MIS-C is debatable. Undoubtedly aneurysms may appear, cases of giant aneurysms have been described^30^. The true prevalence of CAA is unknown though and may be over-estimated, as febrile condition or myocarditis can cause transient coronary dilation too^31^. Both coronary artery aneurysms and dilations were less prevalent in our group than in other reports^4–6,8–10,30^.

It is not established whether race/ethnicity is associated with the severity of the disease^4,6,32^. Non-Hispanic White children comprised only 13–30% of cases in the most numerous MIS-C cohorts and systematic reviews^4–6,8–10,14^. Predominance of White children in our cohort could be considered as a possible explanation of milder clinical presentation with more favourable outcome. However, due to the lack of a control group of other ethnicities in our study, this conclusion should be treated with caution and requires further analysis.

Another distinguishing feature of our cohort was the small proportion of obese children as compared to reports from other countries (6.7% vs. 18–26%)^4,6,9^. This could have possibly resulted from lower obesity prevalence in Polish children (up to 13%)^33^. It is unknown whether obesity is a risk factor for developing MIS-C nor if it is connected to its severity. In our study, we found no association of body mass index (BMI) Z-score or obesity with severity of the disease.

### Severity predictors

We aimed to identify clinical and laboratory features specific to patients who required intensive care. Older age (in line with other reports^4,6^) and male sex were the only demographic characteristics associated with PICU admission. The median time from the first symptoms to hospital admission did not differ significantly for PICU patients. They could be distinguished already at admission by their vital signs: decreased level of consciousness, longer CRT, higher respiratory rate and lower SBP and by the laboratory results. Apart from previously established high inflammatory markers^6,8^, high D-dimers, low eGFR, and presence of the heart injury markers turned out to be severity predictors.

### Limitations

We have noted the following limitations of the presented work. The study relied on voluntary participation, hence a number of MIS-C cases might have been missed or biased by non-random sampling. Some patients meeting the MIS-C criteria may have been misclassified, e.g. due to unequal access to SARS-CoV-2 testing or missing data. Outliers in our data were verified at source whenever possible. Because of the broad MIS-C case definition and highly prevalent COVID-19 in the society, children with alternative diagnoses and coincidental positive SARS-CoV-2 results could be included in our cohort.

Precise epidemiological data on COVID-19 prevalence among age groups in Poland is lacking. We also assumed the risk of contracting the virus in the juvenile population to be homogeneous.

## Conclusion

The severity of MIS-C is not as uniform as it seemed based on previous reports. In particular, race/ethnicity, age, and sex may affect MIS-C phenotype. Consequently, management protocols might not be universally applicable, and should rather be adjusted to the specific population. Patients with altered vital signs and higher inflammatory markers, lower eGFR and markers of heart injury at admission or lower lymphocyte count and albumin concentration during hospitalisation have greater risk of deterioration.

## Methods

### Data sources

The inclusion criteria are presented in Supplementary Table 1. Ethical approval was obtained from the Bioethics Committee at Wroclaw Medical University, Poland (CWN UMW BW: 313/2020). All research was performed in accordance with relevant guidelines and regulations. The Bioethics Committee at Wroclaw Medical University granted waiver of informed consent, as only de-identified data were transmitted and analyzed.

Anonymised patients’ data (demographic, clinical characteristics, laboratory parameters, cardiovascular evaluation results, treatment and outcome data) were extracted from health records and collected through an online questionnaire developed for that purpose. Vital signs and laboratory parameters were obtained at admission and at their respective peaks.

Here we retrospectively report and analyze data covering the period from 4th March 2020 (when the first COVID-19 case was confirmed in Poland) to 20th February 2021. Nine of the presented cases were included in our previous, cursory report^34^.

### MIS-C case definition

We adopted WHO MIS-C case definition^3^, which requires all of the following:

- children 0–18 years old with fever lasting at least 3 days;
- at least two of the following:
  - rash or bilateral conjunctivitis, or mucocutaneous inflammation signs;
  - hypotension defined by a minimal SBP below $70+2\times$ age (in years) mmHg or below 90 mmHg for children older than 10 years^35^;
  - features of myocardial dysfunction, pericarditis, or CAA, based on echocardiographic findings or elevated B-type natriuretic peptide (BNP)/N-terminal-pro-BNP (NT-proBNP), or troponin;
  - evidence of coagulopathy (by international normalised ratio (INR) > 1.1, activated partial thromboplastin time > 40 s or D-dimer > 500 g/ml);
  - acute gastrointestinal problems.
- elevated inflammatory markers: erythrocyte sedimentation rate (ESR) $\ge$ 40 mm/h, CRP $\ge$ 30 mg/l, or procalcitonin $\ge$ 0.5 ng/ml;
- no other apparent microbial cause;
- evidence of COVID-19 (positive RT-PCR, antigen test, or serology), or personal history of COVID-19 or contact with a proven COVID-19 case.

### Standardised study definitions

All standardised study definitions and measures, including laboratory and echocardiographic abnormalities, consciousness level and nutritional status are presented in Supplementary Table 2. They were automatically evaluated by a dedicated software.

#### Laboratory abnormalities

We defined lymphopenia as lymphocyte count $<1.5\times 10^9$/l, anemia according to age-related norms, thrombocytopenia as platelets $< 150 \times 10^9$/l, elevated alanine transaminase as $\ge$ 40 U/l, hyponatremia as serum sodium < 135 mmol/l, hypoalbuminemia as serum albumin < 3.5 g/dl, elevated BNP/NT-proBNP as > 150 ng/ml. A threshold of 50 ng/l defined elevated both T and I troponin. Renal dysfunction was defined by estimated glomerular filtration rate (eGFR) < 90 ml/min/1.73 m$^2$, calculated using the revised Schwartz formula.

#### Echocardiographic abnormalities

Echocardiography results were categorised based on left ventricular EF and coronary artery measurements whenever available. The worst available EF and the largest coronary Z-scores were included. The echocardiography results were assessed by two independent cardiologists. Dilation was defined as a Z-score between 2 and less than 2.5, while aneurysm as Z-score $\ge$ 2.5^36^. Hypotension was defined by a minimal SBP below $70+2\times$age (in years) mmHg or below 90 mmHg for children over 10 years old^35^.

### Clinical definitions

Diagnostic criteria of KD in its typical and atypical form were adapted from AHA guidelines^36^. MAS was defined based on Paediatric Rheumatology International Trials Organization criteria (Supplementary Table 1)^37^. DIC was diagnosed using modified DIC score^38^.

### Statistical methods

We described variables in relation to the sum of cases for which the variable was recorded. We assumed missing values to be distributed randomly and independently from the data, and propagated them through clinical definitions according to Łukasiewicz’s logic. For the assessment of univariate relationships, either direct or divided by sex or age group, and disease severity, we used Cochran–Mantel–Haenszel’s test for two categorical variables and Kruskal–Wallis’s test for categorical-continuous variable pairs. Confidence intervals for incidence values were estimated according to Clopper–Pearson’s method. Significance level of 0.05 and two-sided testing was employed. All statistical analyses were performed in R, version 4.0.3 (R Foundation for Statistical Computing), using coin and GenBinomApps packages. MIS-C incidence was estimated based on the demographic data published by Statistics Poland^39^.

Due to an exploratory nature of this study, we have not adjusted p-values for multiple comparisons.

## Supplementary Information

Supplementary Information.
