# Supplementary material for: Distinct characteristics of multisystem inflammatory syndrome in children in Poland
Source: Sci Rep. 2021 Dec 7;11:23562. doi: 10.1038/s41598-021-02669-2 (PMC8651720; doi:10.1038/s41598-021-02669-2)
Supplement: Supplementary file 1 — Supplementary Information. [file 41598_2021_2669_MOESM1_ESM.pdf]

Distinct characteristics of Multisystem Inflammatory Syndrome in Children in Poland

Supplementary Information

Kamila Maria Ludwikowska[1]*, PhD, Magdalena Okarska-Napierała[2]*, PhD, Natalia Dudek[2], MD, Paweł Tracewski[3], MD, Jacek Kusa[3], PhD, Krzysztof Piwoński[4], MSc, Aneta Afelt[4,5], PhD, Dominik Cysewski[6], MSc, Mateusz Biela[7], MD, Bożena Werner[8], PhD, Teresa Jackowska[9], PhD, Catherine Suski[4], PhD, Miron Bartosz Kursa[4], PhD, Ernest Kuchar[2]**, PhD, Leszek Szenborn[1]**, PhD, MOIS CoR Study Group

*equally contributed
**equally contributed

1) Department of Pediatric Infectious Diseases, Wroclaw Medical University, Chałubińskiego 2-2a, 50-368 Wroclaw, Poland
2) Department of Pediatrics with Clinical Assessment Unit, Medical University of Warsaw, Żwirki i Wigury 61, 02-091 Warsaw, Poland
3) Department of Pediatric Cardiology, Regional Specialist Hospital in Wroclaw, Research and Development Center, Kamieńskiego 73a, 51-124 Wrocław, Poland
4) Interdisciplinary Centre for Mathematical and Computational Modelling, University of Warsaw, Pawinskiego 5A, 02-106, Warsaw, Poland
5) Espace-DEV, IRD - Institut de Recherche pour le Développement, 500 rue Jean-François Breton - 34393 Montpellier cedex 05, France
6) Institute of Biochemistry and Biophysics, Polish Academy of Sciences, Pawinskiego 5A, 02-106, Warsaw, Poland
7) Department of Paediatrics and Rare Disorders, Wroclaw Medical University, Wroclaw, Poland
8) Department of Pediatric Cardiology and General Pediatrics, Medical University of Warsaw, Żwirki i Wigury 61, 02-091 Warsaw, Poland
9) Department of Pediatrics, The Medical Centre of Postgraduate Education, Cegłowska 80, 01-809 Warsaw, Poland

Supplemental Table 1. The Inclusion Criteria for MultiOrgan Inflammatory Syndromes COVID-19 Related Study (MOIS-CoR Study)

**Study inclusion criteria: age, disease severity, timing, diagnosis criterion and exclusion of other causes must be fulfilled.**
Age: 0-18 years
Disease severity: requiring hospitalization
Time frame: since March 4th 2020, ongoing (the paper covers data reported before 20th February 2021)
Diagnosis:<br>Kawasaki disease (KD) OR incomplete (atypical) Kawasaki disease (aKD)<br>OR toxic shock syndrome (TSS) OR macrophage activation syndrome[a] (MAS) OR unspecified inflammatory syndrome
**Kawasaki disease (KD) case definition[b]:**<br>Fever for at least 5 days and 4 from the following symptoms:<br>a) Erythema and cracking of lips, strawberry tongue, and/or erythema of oral and pharyngeal mucosa<br>b) Bilateral bulbar conjunctival injection without exudate<br>c) Rash: maculopapular, diffuse erythroderma, or erythema multiforme-like<br>d) Erythema and oedema of the hands and feet in acute phase and/or periungual desquamation in subacute phase<br>e) Cervical lymphadenopathy (≥1.5 cm diameter)<br><br>**Incomplete (atypical) Kawasaki disease (aKD) case definition:**<br>Fever for at least 5 days and 2 or 3 from the above symptoms OR infant with unexplained fever for at least 7 days AND CRP ≥3 mg/dL and/or ESR ≥40 mm/hr AND<br>1) at least 3 of the following:<br>a) Anaemia for age[c]<br>b) PLT ≥450 $\times 10^{9}$/L after the 7th day of fever<br>c) Albumin ≤3 g/dL<br>d) AL ≥40 U/L T<br>e) WBC count of ≥15 $\times 10^{9}$/L<br>f) Urine ≥10 WBC/hpf<br>OR<br>2) Changes in echocardiogram suggesting KD<br><br>**Toxic shock syndrome (TSS) case definition[d]:**<br>1) Fever AND<br>2) Hypotension[e] AND<br>3) at least two of the following organ systems involvement:<br>a) Gastrointestinal (vomiting, diarrhoea, abdominal pain);<br>b) Muscular (severe myalgia, elevated creatine phosphokinase level);<br>c) Renal (sterile pyuria, elevated creatinine or urea);<br>d) Hepatic (elevated liver enzymes and/or bilirubin level);<br>e) Hematologic (decrease in PLT <100×10^{9}/L);<br>f) Disseminated intravascular coagulation;<br>g) Acute onset of diffuse pulmonary infiltrates and hypoxemia;<br>h) Acute onset of generalized oedema, or pleural or peritoneal effusions with hypoalbuminemia;<br>i) Central nervous system (alterations in consciousness in absence of fever and hypotension)<br><br>**Macrophage activation syndrome (MAS) case definition[f]:**<br>Febrile patient with:<br>1) Ferritin >684 ng/mL AND<br>2) Any two of the following:<br>a) PLT ≤181 $\times 10^{9}$/L<br>b) AST >48 U/L<br>c) Triglycerides >156 mg/dL<br>d) Fibrinogen ≤360 mg/dL<br><br>**Inflammatory syndrome case definition[g]:**<br>1) Fever for at least 3 days AND<br>2) High inflammatory markers (neutrophil count, CRP, ESR, procalcitonin) AND<br>3) Features of at least one organ dysfunction AND<br>4) At least two of the following symptoms:<br>a) Rash or bilateral non-purulent conjunctivitis or mucocutaneous inflammation signs (oral, hands or feet).<br>b) Hypotension or shock.<br>c) Features of myocardial dysfunction, pericarditis, valvulitis, or coronary abnormalities (including echocardiogram findings or elevated troponin/BNP/NT-proBNP),<br>d) Evidence of coagulopathy (by elevated INR, PTT, D-Dimers).<br>e) Acute gastrointestinal problems (diarrhoea, vomiting, or abdominal pain);
Exclusion of other infectious and non-infectious causes that could be responsible for the disease
SARS-CoV-2 testing may be positive or negative.

Abbreviations: ALT, alanine transaminase; AST, aspartate aminotransferase; BNP, brain natriuretic peptide; CRP, C-reactive protein; ESR, erythrocyte sedimentation rate; hpf, high power field; INR, international normalized ratio, NT-proBNP, N-terminal prohormone of brain natriuretic peptide; PLT, platelet count; PTT, partial thromboplastin time; SARS-CoV-2, severe acute respiratory syndrome coronavirus 2; WBC, white blood cells count

[a] DIC diagnosed using modified DIC score[1]

[b] Diagnostic criteria of KD and aKD in its typical and atypical (aKD) form were adapted from American Heart Association guidelines[2]
[c] Haemoglobin norms adapted from Cheng et al.[3]
[d] TSS was defined based on modified criteria by Centers of Disease Control and Prevention[4 5]
[e] Hypotension defined by a minimal systolic blood pressure (sBP) below 70+2×age (in years) mmHg or below 90 mmHg for children over 10 years old[6]
[f] MAS diagnosed using Paediatric Rheumatology International Trials Organization criteria[7]
[g] Adapted from the World Health Organization (WHO) multisystem inflammatory syndrome in children (MIS-C) definition[8]

Supplemental Table 2. Standardised Study Definitions

**Feature**	**Study definition**
Laboratory abnormalities	
Lymphopenia	lymphocytes <1.5 $\times10^9$/L
Anaemia	Hb < age-related norm[a]
Thrombocytopenia	PLT <150 $\times10^9$/L
Elevated alanine transaminase	ALT ≥40 U/L
Hyponatraemia	Na <135 mmol/L
Hypoalbuminaemia	albumin <3.5 g/dL
Elevated BNP/NT-proBNP	BNP or NT-proBNP >150 ng/mL
Elevated T or I troponin	troponin T or I >50 ng/L
Renal dysfunction	eGFR <90 ml/min/1.73 m^2 [b]
Echocardiographic Abnormalities	
Heart dysfunction	EF <55%
Severe heart dysfunction	EF <35%
Coronary artery dilation	Z-score between 2 to 2.5[c]
Coronary artery aneurysm	Z-score ≥2.5[c]
Other measurements	
Level of consciousness	according to AVPU scale
Nutritional status	according to BMI, converted into Z-scores [d]
Obesity	BMI≥95 kg/m2
Disseminated intravascular coagulation (DIC)	At least 5 points in scale:<br>1) PLT <50 $\times10^9$/l=2 pt; 50-100$\times10^9$/l=1 pt; >100$\times10^9$/l=0 pt<br>2) D-dimers >2 mg/l=3 pt; 0,5-2 mg/l= 2 pt; <0,5 mg/l= 0 pt<br>3) INR >1.6=2 pt; 1,3-1,6=1 pt; <1,3=0 pt<br>4) fibrinogen min <1 g/l= 1 pt; ≥1 g/l= 0 pt

Abbreviations: ALT, alanine transaminase; BMI, body-mass index; BNP, brain natriuretic peptide; EF, left ventricle ejection fraction; Hb, haemoglobin; INR, international normalized ratio; Na, sodium; NT-proBNP, N-terminal prohormone of brain natriuretic peptide; PLT, platelet count

[a] Haemoglobin norms adapted from Cheng et al.[3]

[b] Assessed with Schwartz formula[9]

[c] According to the American Heart Association definitions for Kawasaki disease;[2] Z-score was calculated using Dallaire equation[10] or Boston Children's Hospital Z-score calculator,[11] depending on the body surface area. The worst available EF and the largest coronary Z-scores were included. The echography results were assessed by two independent cardiologists.

[d] Assessed using the body-mass index (BMI), converted into Z-scores based on the WHO reference standards for children younger than 5 years[12] and national reference standards for older children[13]

Supplemental Table 3. SARS-CoV-2 laboratory tests and anamnesis

	Cases	Percent of tested	Percent of all
Contact with a confirmed COVID-19 case preceding MIS-C	120	51.9%	43.8%
Confirmed SARS-CoV-2 infection preceding MIS-C	24	9.8%	8.8%
Positive SARS-CoV-2 RT-PCR result at the time of MIS-C	29	12.6%	10.6%
Positive antibodies against SARS-CoV-2 at the time of MIS-C	241	94.5%	88.0%

Abbreviations: COVID-19, coronavirus disease 2019; MIS-C, multisystem inflammatory syndrome in children; RT-PCR, real-time transcription polymerase chain reaction; SARS-CoV-2, severe acute respiratory syndrome coronavirus 2

Supplemental Table 4. Demographic and clinical characteristics, management and outcome of MIS-C cohort

	Overall (N=274)	<5 y (N=64)	5-12 y (N=140)	12-18 y (N=70)	p	p sex-adjusted	PICU patients (N=23)	non-PICU patients (N=251)	p	p age-adjusted	p sex-adjusted
	n or med (% or IQR)	n or med (% or IQR)	n or med (% or IQR)	n or med (% or IQR)			n or med (% or IQR)	n or med (% or IQR)			
Male sex	172 (63%)	30 (47%)	91 (65%)	51 (73%)	<0.01	1	17 (74%)	155 (62%)	0.2	0.5	1
Age (years)	8.8 (5.2-12.1)	3.2 (2.1-4.3)	8.7 (6.6-10.5)	13.6 (12.8-14.8)	<0.01	<0.01	11.2 (10.1-12.6)	8.4 (5.0-11.9)	<0.01	0.03	<0.01
BMI Z-score	0.1 (-0.8-0.9)	-0.3 (-0.9-0.7)	0.1 (-0.8-1.1)	0.5 (-0.3-0.8)	0.07	0.04	0.7 (-0.3-0.9)	0.0 (-0.8-0.9)	0.2	0.3	0.2
**Comorbidities**	38 (18%)	7 (14%)	21 (20%)	10 (19%)	0.7	0.7	4 (21%)	34 (18%)	0.7	0.8	0.7
Obesity	16 (7%)	2 (4%)	11 (9%)	3 (5%)	0.3	0.3	3 (14%)	13 (6%)	0.1	0.2	0.09
Asthma	11 (4%)	2 (3%)	6 (4%)	3 (4%)	0.9	0.9	1 (5%)	10 (4%)	0.9	1	0.9
Chronic neurological issue	6 (2%)	2 (3%)	3 (2%)	1 (1%)	0.8	0.7	-	6 (2%)	0.5	0.5	0.4
Other	9 (4%)	2 (4%)	4 (3%)	3 (6%)	0.8	0.8	-	9 (4%)	0.3	0.3	0.3
**Symptoms and signs**											
**Fever length (days)**	7.0 (6.0-9.0)	7.0 (6.0-8.0)	7.5 (6.0-9.0)	7.0 (6.0-9.0)	0.3	0.3	8.0 (7.0-10.0)	7.0 (6.0-9.0)	0.03	.06	0.04
**Mucocutaneous and lymph nodes**	262 (97%)	59 (95%)	138 (99%)	65 (94%)	0.2	0.2	22 (96%)	240 (97%)	0.8	0.8	0.7
Rash	218 (83%)	53 (84%)	118 (87%)	47 (71%)	0.02	0.02	15 (71%)	203 (84%)	0.2	0.2	0.2
Erythema at BCG site	1 (0%)	1 (2%)	-	-	0.2	0.4	-	1 (0%)	0.8	0.9	0.8
Conjunctivitis	207 (78%)	47 (76%)	109 (81%)	51 (75%)	0.6	0.6	14 (67%)	193 (79%)	0.2	0.2	0.2
Hands/feet swelling or erythema	142 (55%)	39 (64%)	75 (56%)	28 (45%)	0.1	0.2	15 (68%)	127 (54%)	0.2	0.1	0.1
Oral inflammation	173 (66%)	43 (68%)	96 (72%)	34 (52%)	0.02	0.02	14 (67%)	159 (66%)	0.9	0.8	1
Cervical lymphadenopathy	98 (38%)	30 (48%)	46 (36%)	22 (32%)	0.1	0.07	7 (33%)	91 (38%)	0.7	0.9	0.6
**Gastrointestinal**	250 (93%)	56 (90%)	132 (95%)	62 (90%)	0.3	0.3	21 (91%)	229 (93%)	0.8	0.8	0.6
Abdominal pain	222 (85%)	42 (76%)	120 (88%)	60 (87%)	0.1	0.2	21 (91%)	201 (85%)	0.4	0.5	0.5
Nausea	162 (62%)	28 (47%)	100 (75%)	34 (51%)	<0.01	<0.01	16 (70%)	146 (61%)	0.4	0.5	0.5
Diarrhoea	164 (62%)	36 (59%)	84 (61%)	44 (65%)	0.8	0.9	18 (78%)	146 (60%)	0.09	0.1	0.1
**Upper respiratory**	98 (38%)	30 (54%)	42 (31%)	26 (39%)	0.02	0.03	10 (45%)	88 (38%)	0.5	0.3	0.4
Sore throat	86 (34%)	25 (47%)	38 (28%)	23 (35%)	0.05	0.05	8 (36%)	78 (34%)	0.8	0.6	0.8
Rhinitis	23 (9%)	10 (16%)	7 (5%)	6 (9%)	0.05	0.09	2 (10%)	21 (9%)	0.9	0.7	0.8
**Lower Respiratory**	128 (50%)	23 (40%)	64 (48%)	41 (63%)	0.04	0.05	18 (86%)	110 (47%)	<0.01	<0.01	<0.01
Chest pain	48 (19%)	3 (6%)	19 (14%)	26 (39%)	<0.01	<0.01	5 (25%)	43 (18%)	0.5	1	0.5
Cough	74 (28%)	17 (28%)	33 (25%)	24 (35%)	0.3	0.2	7 (32%)	67 (28%)	0.7	0.8	0.6
Dyspnoea	63 (24%)	8 (13%)	36 (27%)	19 (28%)	0.08	0.1	14 (67%)	49 (20%)	<0.01	<0.01	<0.01
**Neurological**	220 (86%)	54 (86%)	112 (85%)	54 (87%)	0.9	0.9	20 (100%)	200 (84%)	0.06	0.06	0.05
Lethargy	151 (59%)	36 (61%)	88 (66%)	27 (44%)	0.02	0.02	19 (90%)	132 (57%)	<0.01	<0.01	<0.01
Irritability	108 (42%)	40 (65%)	52 (39%)	16 (25%)	<0.01	<0.01	6 (27%)	102 (43%)	0.2	0.4	0.2
Headache	112 (46%)	16 (32%)	67 (51%)	29 (47%)	0.07	0.04	8 (38%)	104 (47%)	0.4	0.3	0.5

	Overall (N=274)	<5 y (N=64)	5-12 y (N=140)	12-18 y (N=70)	p	p sex-adjusted	PICU patients (N=23)	non-PICU patients (N=251)	p	p age-adjusted	p sex-adjusted
Meningeal signs	27 (10%)	6 (10%)	15 (11%)	6 (9%)	0.9	0.9	2 (9%)	25 (11%)	0.8	0.8	0.8
Seizures	3 (1%)	2 (3%)	1 (1%)	-	0.2	0.06	-	3 (1%)	0.6	0.7	0.6
Muscle hypotension	30 (11%)	5 (8%)	16 (12%)	9 (14%)	0.6	0.7	5 (24%)	25 (10%)	0.07	0.1	0.07
Nerve paralysis	2 (1%)	1 (2%)	1 (1%)	-	0.6	0.6	-	2 (1%)	0.7	0.7	0.7
Nerve paresis	2 (1%)	-	2 (1%)	-	0.4	0.4	-	2 (1%)	0.7	0.7	0.7
Smell loss	8 (3%)	1 (2%)	2 (1%)	5 (8%)	0.05	0.02	1 (5%)	7 (3%)	0.7	0.9	0.6
Taste loss	7 (3%)	1 (2%)	2 (1%)	4 (6%)	0.2	0.08	1 (5%)	6 (3%)	0.6	0.7	0.5
Photophobia	28 (11%)	8 (14%)	16 (12%)	4 (6%)	0.4	0.4	-	28 (12%)	0.1	0.1	0.1
Skin hyperesthesia	87 (34%)	29 (48%)	46 (35%)	12 (19%)	<0.01	<0.01	8 (36%)	79 (34%)	0.8	0.4	0.7
**Cardiovascular**	139 (58%)	16 (33%)	79 (63%)	44 (69%)	<0.01	<0.01	22 (100%)	117 (54%)	<0.01	<0.01	<0.01
Hypotension[a]	99 (41%)	7 (14%)	57 (46%)	35 (51%)	<0.01	<0.01	18 (86%)	81 (36%)	<0.01	<0.01	<0.01
Coronary dilation or aneurysm[b]	21 (8%)	6 (11%)	10 (7%)	5 (8%)	0.8	0.5	3 (14%)	18 (8%)	0.3	0.3	0.4
EF<55%	58 (23%)	3 (5%)	31 (23%)	24 (39%)	<0.01	<0.01	14 (64%)	44 (19%)	<0.01	<0.01	<0.01
Pericardial effusion	24 (9%)	5 (9%)	15 (11%)	4 (6%)	0.6	0.5	3 (14%)	21 (9%)	0.5	0.5	0.6
**Musculo-osteoarticular**	111 (44%)	17 (32%)	58 (44%)	36 (55%)	0.04	0.03	8 (42%)	103 (44%)	0.8	0.6	0.9
Muscle pain	103 (41%)	15 (29%)	53 (40%)	35 (54%)	0.02	0.01	8 (42%)	95 (41%)	0.9	0.8	0.9
Arthralgia	50 (19%)	6 (10%)	29 (21%)	15 (24%)	0.1	0.08	4 (20%)	46 (19%)	0.9	0.8	0.9
Arthritis	12 (5%)	1 (2%)	6 (4%)	5 (8%)	0.2	0.2	1 (5%)	11 (5%)	1	0.8	1
Scrotum/labia swelling	26 (10%)	8 (13%)	12 (9%)	6 (10%)	0.6	0.2	3 (14%)	23 (10%)	0.5	0.4	0.7
**Clinical presentation**											
KD[c]	170 (67%)	43 (70%)	94 (71%)	33 (54%)	0.06	0.09	18 (86%)	152 (65%)	0.05	0.03	0.04
MAS[d]	59 (22%)	4 (6%)	30 (22%)	25 (36%)	<0.01	<0.01	9 (43%)	50 (20%)	0.02	0.08	0.02
DIC[e]	42 (17%)	5 (9%)	22 (17%)	15 (22%)	0.1	0.06	5 (22%)	37 (16%)	0.5	0.7	0.4
**Therapy and outcome**											
Admission since onset (days)	5.0 (4.0-6.0)	5.0 (4.0-5.0)	5.0 (4.0-6.0)	5.0 (4.0-6.5)	0.5	0.5	6.0 (4.0-7.0)	5.0 (4.0-6.0)	0.08	0.1	0.09
Intensive care	23 (8%)	1 (2%)	13 (9%)	9 (13%)	0.05	0.08	23 (100%)	-	<0.01	<0.01	<0.01
Mechanical ventilation	10 (4%)	-	7 (5%)	3 (5%)	0.2	0.2	10 (43%)	-	<0.01	<0.01	<0.01
Oxygen supplementation	61 (23%)	5 (8%)	41 (31%)	15 (23%)	<0.01	<0.01	17 (77%)	44 (18%)	<0.01	<0.01	<0.01
No immunomodulatory agent	8 (3%)	3 (5%)	3 (2%)	2 (3%)	0.6	0.4	-	8 (3%)	0.4	0.4	0.3
IVIG	238 (93%)	55 (90%)	122 (95%)	61 (91%)	0.3	0.3	20 (100%)	218 (92%)	0.2	0.2	0.2
GCS	143 (67%)	27 (56%)	74 (69%)	42 (71%)	0.2	0.2	17 (89%)	126 (64%)	0.03	0.04	0.03
IVIG and GCS	133 (62%)	24 (50%)	69 (65%)	40 (67%)	0.1	0.2	16 (89%)	117 (60%)	0.01	0.03	0.02
Other immunomodulatory agents	3 (1%)	1 (2%)	2 (2%)	-	0.6	0.7	-	3 (2%)	0.6	0.7	0.6
ASA	226 (85%)	51 (81%)	119 (88%)	56 (84%)	0.4	0.4	18 (90%)	208 (85%)	0.5	0.6	0.5
Heparin	91 (38%)	14 (25%)	45 (38%)	32 (49%)	0.02	0.02	18 (100%)	73 (33%)	<0.01	<0.01	<0.01

	Overall (N=274)	<5 y (N=64)	5-12 y (N=140)	12-18 y (N=70)	p	p sex-adjusted	PICU patients (N=23)	non-PICU patients (N=251)	p	p age-adjusted	p sex-adjusted
Warfarin	1 (0%)	1 (2%)	-	-	0.2	0.1	-	1 (1%)	0.8	0.9	0.7
Complete recovery at discharge	218 (93%)	55 (95%)	115 (94%)	48 (87%)	0.2	0.2	18 (86%)	200 (93%)	0.2	0.3	0.2

Abbreviations: ASA, acetylsalicylic acid; BCG, Bacillus Calmette–Guérin vaccine; BMI, body mass index; DIC, disseminated intravascular coagulation; EF, ejection fraction; GCS, glucocorticoids; IQR, interquartile range; IVIG, intravenous immunoglobulin; KD, Kawasaki disease; MAS, macrophage activation syndrome; med, median; MIS-C, multisystem inflammatory syndrome in children; PICU, paediatric intensive care unit; y, years old

[a] Defined by a minimal systolic blood pressure below 70+2×age (in years) mmHg or below 90 mmHg for children over 10 years old[6]

[b] Dilation was defined as a Z-score between 2 to 2.5, while aneurysm as Z-score ≥2.5[2 10 11]

[c] Diagnostic criteria of KD in its typical and atypical (aKD) form were adapted from American Heart Association guidelines[2]

[d] MAS was diagnosed based on Paediatric Rheumatology International Trials Organization criteria[7]

[e] DIC was diagnosed using modified DIC score[1]

Supplemental Table 5. Vital signs and laboratory results of MIS-C cohort at admission and at respective peaks.

	All (274)	PICU (23)	non-PICU (251)	P	P|Age	P|Sex
CRT >2s adm	30 (13%)	8 (40%)	22 (11%)	<0.01	<0.01	<0.01
CRT max >2s	32 (15%)	10 (56%)	22 (11%)	<0.01	<0.01	<0.01
Saturation adm [%]	98.0 (96.0-99.0)	97.0 (95.0-98.0)	98.0 (96.0-99.0)	0.09	0.08	0.09
Saturation min [%]	96.0 (92.0-98.0)	92.0 (89.8-97.0)	96.0 (93.0-98.0)	0.02	0.04	0.02
Respiratory rate adm [1/min]	20.0 (18.0-25.0)	30.0 (20.0-45.0)	20.0 (18.0-25.0)	0.01	<0.01	<0.01
Respiratory rate max [1/min]	25.0 (20.0-30.0)	40.0 (30.0-49.5)	24.0 (20.0-30.0)	<0.01	<0.01	<0.01
Heartrate adm [1/min]	120.0 (100.0-133.0)	127.0 (110.0-140.0)	120.0 (100.0-133.0)	0.3	0.08	0.2
Heartrate max [1/min]	132.5 (118.0-150.0)	135.0 (120.0-160.0)	132.0 (118.0-146.5)	0.2	0.06	0.2
SBP adm [mmHg]	100.0 (90.0-110.0)	89.0 (78.8-99.5)	100.0 (91.0-110.0)	<0.01	<0.01	<0.01
SBP min [mmHg]	88.0 (78.0-96.0)	74.0 (62.5-82.5)	89.0 (80.0-96.8)	<0.01	<0.01	<0.01
Non-alert AVPU adm	17 (6%)	5 (22%)	12 (5%)	<0.01	<0.01	<0.01
Non-alert AVPU min	38 (15%)	8 (44%)	30 (13%)	<0.01	<0.01	<0.01
WBC adm [10^9/l]	9.6 (6.6-13.3)	11.6 (7.1-19.1)	9.6 (6.6-12.8)	0.1	0.1	0.1
WBC min [10^9/l]	6.7 (5.0-9.0)	6.0 (5.3-9.2)	6.7 (5.0-9.0)	0.9	0.8	0.9
WBC max [10^9/l]	14.5 (10.9-19.7)	19.8 (13.1-28.4)	14.3 (10.5-19.1)	<0.01	<0.01	<0.01
Lymphocytes adm [10^9/l]	1.0 (0.7-1.8)	0.8 (0.6-1.0)	1.1 (0.7-1.8)	0.05	0.3	0.05
Lymphocytes min [10^9/l]	1.0 (0.6-1.8)	0.6 (0.5-0.9)	1.0 (0.6-1.8)	<0.01	0.07	<0.01
Hemoglobin adm [g/dl]	11.7 (10.7-12.7)	11.1 (10.3-12.1)	11.8 (10.7-12.7)	0.1	0.02	0.1
Hemoglobin min [g/dl]	10.3 (9.4-11.2)	9.7 (8.8-10.7)	10.3 (9.4-11.2)	0.06	<0.01	0.05
Platelets adm [10^9/l]	176.0 (127.0-248.0)	153.0 (121.0-187.5)	178.0 (128.5-255.8)	0.08	0.1	0.07
Platelets min [10^9/l]	160.0 (109.8-230.8)	135.5 (93.8-182.5)	163.5 (111.0-243.2)	0.08	0.09	0.06
CRP adm [mg/l]	140.0 (83.7-194.9)	242.0 (123.3-289.0)	133.4 (78.8-187.2)	<0.01	<0.01	<0.01
CRP max [mg/l]	166.3 (94.4-242.1)	264.6 (206.7-309.4)	161.4 (93.3-226.2)	<0.01	<0.01	<0.01
Procalcitonin adm [ng/ml]	2.5 (1.0-6.9)	13.2 (2.1-51.2)	2.3 (0.9-6.2)	<0.01	<0.01	<0.01
Procalcitonin max [ng/ml]	4.3 (1.3-12.9)	17.0 (10.2-30.7)	3.5 (1.2-10.0)	<0.01	<0.01	<0.01
ESR [mm] adm	44.0 (31.0-66.0)	78.5 (73.0-81.2)	44.0 (30.0-65.0)	0.03	0.02	0.02
ESR [mm] max	57.0 (37.8-80.0)	77.0 (52.5-82.5)	55.0 (37.0-78.0)	0.4	0.4	0.4
Ferritin adm [ng/ml]	331.0 (197.9-622.4)	671.0 (475.9-1052.8)	317.1 (186.2-533.8)	<0.01	<0.01	<0.01
Ferritin max [ng/ml]	402.2 (217.9-672.2)	671.0 (559.2-1113.4)	367.9 (207.3-616.5)	<0.01	<0.01	<0.01
Triglycerides adm [mg/dl]	147.0 (123.0-218.9)	181.0 (148.5-266.5)	145.0 (119.0-213.0)	0.05	0.04	0.05

	All (274)	PICU (23)	non-PICU (251)	P	P|Age	P|Sex
Triglycerides max [mg/dl]	172.5 (129.0-256.2)	194.0 (169.0-359.0)	167.0 (125.0-246.0)	0.07	0.05	0.07
D-dimers adm [mg/l]	2.6 (1.5-4.6)	3.9 (2.5-5.7)	2.5 (1.4-4.4)	0.01	<0.01	<0.01
D-dimers max [mg/l]	3.8 (2.0-6.3)	5.7 (3.6-8.2)	3.7 (2.0-6.2)	0.03	0.05	0.02
INR adm	1.2 (1.1-1.4)	1.3 (1.2-1.4)	1.2 (1.1-1.3)	0.3	0.5	0.2
INR max	1.2 (1.1-1.4)	1.3 (1.2-1.4)	1.2 (1.1-1.4)	0.1	0.2	0.1
AlAT adm [U/l]	24.0 (16.0-40.1)	35.0 (16.0-92.0)	23.0 (15.5-40.0)	0.05	0.06	0.05
AlAT max [U/l]	33.3 (21.0-65.8)	55.0 (32.0-122.0)	33.0 (20.0-59.2)	<0.01	0.03	<0.01
AST adm [U/l]	32.1 (25.0-51.5)	37.0 (25.0-59.0)	32.0 (24.5-51.0)	0.3	0.3	0.3
AST max [U/l]	43.0 (30.0-65.0)	57.0 (44.0-108.0)	42.0 (30.0-63.5)	<0.01	0.01	<0.01
LDH adm [U/l]	278.0 (228.0-334.1)	264.5 (238.0-425.5)	281.0 (224.0-330.0)	0.7	0.6	0.6
LDH max [U/l]	295.0 (239.0-353.0)	265.0 (246.0-344.0)	296.0 (239.0-353.0)	0.9	0.9	0.9
CK adm [U/l]	51.0 (37.0-92.0)	22.4 (13.2-30.9)	52.0 (38.0-93.0)	0.01	<0.01	0.02
CK max [U/l]	51.0 (37.0-85.0)	27.9 (24.0-38.0)	56.5 (37.2-92.8)	0.01	<0.01	0.01
Lactates adm [mmol/l]	2.0 (1.6-3.0)	2.1 (1.5-4.2)	2.0 (1.6-2.9)	0.4	0.4	0.4
Lactates max [mmol/l]	2.5 (1.8-3.5)	3.5 (2.8-5.0)	2.5 (1.8-3.4)	0.01	0.02	0.01
Sodium adm [mmol/l]	135.0 (132.0-137.0)	134.0 (132.0-137.8)	135.0 (132.0-137.0)	1	0.9	0.8
Sodium min [mmol/l]	133.6 (131.0-135.9)	133.0 (130.0-135.0)	133.8 (131.0-135.9)	0.5	0.6	0.6
Albumins adm [g/dl]	3.3 (2.8-3.7)	2.8 (2.4-3.4)	3.3 (2.8-3.7)	0.02	<0.01	0.01
Albumins min [g/dl]	2.8 (2.5-3.3)	2.5 (2.3-2.7)	2.8 (2.5-3.3)	<0.01	<0.01	<0.01
Troponin elevated adm	62 (28%)	17 (77%)	45 (22%)	<0.01	<0.01	<0.01
Troponin elevated max	92 (51%)	18 (86%)	74 (47%)	<0.01	<0.01	<0.01
BNP/NT-proBNP elevated adm	171 (86%)	16 (89%)	155 (85%)	0.7	0.6	0.7
BNP/NT-proBNP elevated	204 (91%)	20 (100%)	184 (90%)	0.1	0.2	0.1
Creatinine adm [mg/dl]	0.5 (0.4-0.7)	0.8 (0.6-1.4)	0.5 (0.4-0.6)	<0.01	<0.01	<0.01
Creatinine max [mg/dl]	0.5 (0.4-0.7)	0.8 (0.6-1.4)	0.5 (0.4-0.7)	<0.01	<0.01	<0.01
eGFR adm [ml/min/1.73m^2]	110.1 (86.2-134.3)	77.1 (50.8-89.5)	113.1 (90.5-134.8)	<0.01	<0.01	<0.01
eGFR min [ml/min/1.73m^2]	104.9 (82.0-126.6)	73.0 (45.4-88.0)	107.4 (85.4-128.8)	<0.01	<0.01	<0.01

Binary data given as count (per-cent), and numerical data as median (interquartile range). Values at admission are marked with adm, lowest obtained with min, while highest with max. Troponin is considered elevated at >50 ng/l, while BNP/NT-proBNP at >150 ng/ml.
Abbreviations: AlAT, alanine transaminase; AST, aspartate aminotransferase; BNP, brain natriuretic peptide; CK, creatine kinase; CRP C-reactive protein; CRT, capillary refill time; eGFR, estimated glomerular filtration rate ; ESR, erythrocyte sedimentation rate; INR, international normalized ratio, LDH, lactate dehydrogenase ; NT-proBNP, N-terminal prohormone of brain natriuretic peptide;   SBP, systolic blood pressure ; WBC , white blood cells count

Increase of the Kawasaki Disease cases in two reporting sites in years 2015-2021

Cases of Kawasaki disease (KD) and atypical KD (aKD) were extracted from historical medical records from the Pediatric Infectious Disease Department, Wrocław, Poland and the Department of Pediatrics with Clinical Assessment Unit, Warsaw, Poland. Fulfillment of KD/aKD criteria according to the American Heart Association[2] was verified independently by KML, MON and ND.

Supplemental Table 6 Kawasaki Disease (typical and atypical form) hospitalizations per year in 2015-2020/2021 in the Pediatric Infectious Disease Department, Wrocław, Poland and the Department of Pediatrics with Clinical Assessment Unit, Warsaw, Poland.

	Pre-pandemic data					During the pandemic study period
Year	2015	2016	2017	2018	2019	04.03.2020-20.02.2021
No of KD/aKD Wroclaw	9	7	2	13	12	18
No of KD/aKD Warsaw	5	13	15	19	15	52
Summary	14	20	17	32	27	70
Median no per year	18.5					

Abbreviations: KD, Kawasaki Disease; aKD atypical Kawasaki Disease; No, number

Supplementary References:

1. Levi M, Toh CH, Thachil J, et al. Guidelines for the diagnosis and management of disseminated intravascular coagulation. British Committee for Standards in Haematology. Br J Haematol 2009;145(1):24-33. doi: 10.1111/j.1365-2141.2009.07600.x [published Online First: 2009/02/19]
2. McCrindle BW, Rowley AH, Newburger JW, et al. Diagnosis, Treatment, and Long-Term Management of Kawasaki Disease: A Scientific Statement for Health Professionals From the American Heart Association. Circulation 2017;135(17):e927-e99. doi: 10.1161/cir.0000000000000484 [published Online First: 2017/03/31]
3. Cheng CK, Chan J, Cembrowski GS, et al. Complete blood count reference interval diagrams derived from NHANES III: stratification by age, sex, and race. Lab Hematol 2004;10(1):42-53. doi: 10.1532/lh96.04010 [published Online First: 2004/04/09]
4. Centers for Disease Control and Prevention. Streptococcal Toxic Shock Syndrome (STSS) (Streptococcus pyogenes) 2010 Case Definition. Available: https://wwwn.cdc.gov/nndss/conditions/streptococcal-toxic-shock-syndrome/case-definition/2010/ [Accessed 2021/02/01].
5. Centers for Disease Control and Prevention. Toxic Shock Syndrome (Other Than Streptococcal) (TSS) 2011 Case Definition. Available: https://wwwn.cdc.gov/nndss/conditions/toxic-shock-syndrome-other-than-streptococcal/case-definition/2011/ [Accessed 2021/02/01]..

6. Kleinman ME, Chameides L, Schexnayder SM, et al. Pediatric advanced life support: 2010 American Heart Association Guidelines for Cardiopulmonary Resuscitation and Emergency Cardiovascular Care. *Pediatrics* 2010;126(5):e1361-99. doi: 10.1542/peds.2010-2972D [published Online First: 2010/10/20]
7. Minoia F, Bovis F, Davì S, et al. Development and initial validation of the MS score for diagnosis of macrophage activation syndrome in systemic juvenile idiopathic arthritis. Ann Rheum Dis 2019;78(10):1357-62. doi: 10.1136/annrheumdis-2019-215211 [published Online First: 2019/07/13]
8. World Health Organization. Multisystem inflammatory syndrome in children and adolescents with COVID-19. Available: https://www.who.int/news-room/commentaries/detail/multisystem-inflammatory-syndrome-in-children-and-adolescents-with-covid-19 [Accessed 2021/02/27].
9. Schwartz GJ, Work DF. Measurement and estimation of GFR in children and adolescents. Clin J Am Soc Nephrol 2009;4(11):1832-43. doi: 10.2215/cjn.01640309 [published Online First: 2009/10/13]
10. Dallaire F, Dahdah N. New equations and a critical appraisal of coronary artery Z scores in healthy children. J Am Soc Echocardiogr 2011;24(1):60-74. doi: 10.1016/j.echo.2010.10.004 [published Online First: 2010/11/16]
11. Boston Children's Hospital Heart Centre. Z-Score Calculator. Available: https://zscore.chboston.org/ [Accessed 2021/02/28].
12. World Health Organization. Body mass index-for-age (BMI-for-age). Available: https://www.who.int/toolkits/child-growth-standards/standards/body-mass-index-for-age-bmi-for-age [Accessed 2021/02/21].
13. Kułaga Z, Litwin M, Tkaczyk M, et al. Polish 2010 growth references for school-aged children and adolescents. Eur J Pediatr 2011;170(5):599-609. doi: 10.1007/s00431-010-1329-x [published Online First: 2010/10/26]
